# The aldehyde dehydrogenase 2 polymorphisms on neuropsychological performance in bipolar II disorder with or without comorbid anxiety disorder

**DOI:** 10.1371/journal.pone.0192229

**Published:** 2018-02-09

**Authors:** Ru-Band Lu, Yun-Hsuan Chang, Tzu-Yun Wang, Sheng-Yu Lee, Po See Chen, Yen Kuang Yang

**Affiliations:** 1 Department of Psychiatry, National Cheng Kung University Hospital, College of Medicine, National Cheng Kung University, Tainan, Taiwan; 2 Department of Psychology, College of Medical and Health Science, Asia University, Taichung, Taiwan; 3 Department of Medical Research, China Medical University Hospital, China Medical University, Taichung, Taiwan; 4 Department of Psychiatry, National Cheng Kung University Hospital, Dou-Liou Branch, Yunlin, Taiwan; 5 Department of Psychiatry, Kaohsiung Veteran’s General Hospital, Kaohsiung, Taiwan; 6 Department of Psychiatry, College of Medicine, National Yang-Ming University, Taipei, Taiwan; 7 Department of Psychiatry, Faculty of Medicine, Kaohsiung Medical University Kaohsiung, Taiwan; 8 Institute of Behavioral Medicine, College of Medicine, National Cheng Kung University, Tainan, Taiwan; Peking University, CHINA

## Abstract

Anxiety disorders (ADs), the most common comorbid illnesses with bipolar disorder (BP) has been reported to associate with dopamine system. Dopamine, metabolized to 3,4-dihydroxyphenylacetic acid (DOPAC) by aldehyde dehydrogenase 2 (ALDH2), and the distribution of the ALDH2*1/*1, and ALDH2*1/*2+ALDH*2/*2 alleles in the Han Chinese general population is relatively equal. The association between dopamine metabolic enzymes and cognitive performance in patients with bipolar II disorder (BP-II) comorbid with AD is unclear. This study proposed to explore the role of *ALDH2* polymorphisms on neuropsychological performance between BP-II comorbid with or without AD. One hundred ninety-seven BP-II patients with and without a comorbid AD were recruited and compared with 130 healthy controls (HCs). A polymerase chain reaction and a restriction fragment length polymorphism analysis were used to determine genotypes for *ALDH2*, and study participants underwent neuropsychological tests. An interaction between AD comorbidity and the *ALDH2* polymorphisms was found in different domain of cognitive dysfunction in the BP-II patients. The *ALDH2* polymorphisms might have different effects on the neuropsychological performance of BP-II patients with and without comorbid AD.

## Introduction

Patients with bipolar disorder (BP) often complain about attention problems and an impaired ability to think and concentrate, which is one criterion for the diagnosis [1]. Anxiety disorder (AD) is a common comorbidity with BP [2–7], and a growing number of studies have reported a high lifetime prevalence of AD comorbid with BP (BP^+AD^). People with anxiety disorders (ADs) also present with impaired attention and concentration. With such cognitive impairment would negatively affect a patient’s drug adherence, treatment effects, and prognosis [8]. These cognitive difficulties especially are related to verbal memory, might help to explain their impairment in daily functioning, even during remission. Therefore, studying how attention and memory functioning is affected by comorbidity should be helpful for understanding and improving the psychosocial functioning of BP patients with comorbidity.

Several studies have reported cognitive deficits in BP patients, around 30–50% of BP patients are unable to restore their premorbid psychosocial functioning [9]. Other evidence showed that the psychosocial function impairment was highly associated with the cognitive dysfunctioning. Martínez-Arán et al. (2004) reported that even in euthymic states, BP patients had lower scores on executive function and verbal memory tests than did healthy controls, and that neuropsychological functioning and the global assessment of functioning were highly correlated [10].

Higher than 50% prevalence (51.2%) reported by the National Institute of Mental Health (NIMH) [11, 12], and a 31% of the first 500 enrolled patietns in the Systematic Treatment Enhancement Program for Bipolar Disorder (STEP-BD) [12] were reported to have comorbidity with anxiety disorders. Another issue of comorbidity in bipolar disordered patients is recognition and prognosis. BP^+AD^ patients have a higher risk of suicidal behavior [13], substance abuse [12, 14, 15], poorer psychosocial performance [16, 17], and a more frequent family history of mental illness than do BP patients without comorbid AD (BP^‒AD^) [18].

Studies have reported more than one aspect of impairment in patients with BP-II than with BP-I, including impaired working memory [8, 19, 20], psychomotor speed [19, 20], verbal learning, and verbal memory [19]. In addition, BP-II^+AD^ patients had neuropsychological declines in visual immediate memory, visual delayed memory, working memory, and psychomotor speed that were more severe and more widespread than were those for BP-I^+AD^ patients.

Wu et al. (2011) [21] reported impairments in Auditory Immediate and Auditory Delayed memory, but not in Auditory Recognition Delayed memory for BP-II^+AD^ patients, implying a possible dysfunction in retrieving verbal information for the BP-II^+AD^ [22] even, during their inter-episodes. Moreover, a significant poorer dysfunctional frontal lobe and partial executive dysfunction was reported [23]. Furthermore, an association between BP and the disruption of subcortical circuitry, in regulating mood and anxious behaviors has been reported in several neuroimaging studies [24–26]. A smaller volume of these brain areas have been reported in BP patients and AD patients compared to healthy controls [26–29]. In addition, a smaller hippocampal volume was found in BP-II^+AD^ patients than in BP-II^-AD^ patients [30], implying an over-activity of the hypothalamic-pituitary-adrenocortical axis induced by anxiety for a long time and caused impairments of the hippocampus. Thus, the relationship between anxiety and memory has attracted the attention of researchers [31]. So far, the mechanism and the relationship between AD comorbidity and profile of neuropsychological impairments in the BP-II remain unclear.

A primary relationship between BP-II^+AD^ and the dopamine system has been reported [11]; the dopamine system might explain the difference between BP-II^+AD^ and BP-II^−AD^. Previously, An association between DRD2 Taq-I A1/A1 and the ALDH2*1*1 genotypes in BP-II^-AD^ has been reported [32], suggesting a possible genetic distinguish between BPII comorbid with AD or not.

Aldehyde dehydrogenase (ALDH) is an important enzyme in the dopamine metabolic pathway. Aldehyde dehydrogenase 2 (ALDH2) is a mitochondrial isozyme that has been proposed to be responsible for DOPAL (3,4-dihydroxyphenylacetaldehyde) metabolism to DOPAC (3,4-dihydroxyphenylacetic acid) [33, 34]. The *ALDH2* gene (rs671), the functional single nucleotide polymorphism (SNP) in exon 12, which yields two alleles: ALDH2*1 and ALDH2*2 [35, 36]. Based on the reduced acetaldehyde metabolic effect of two genotypes, the ALDH2*1/*1-encoded enzyme is an active form in the metabolism of acetaldehyde, but the enzymes encoded by the ALDH2*1/*2 and *2/*2 polymorphisms (ALDH2*2 allele) are partially or (1/10 metabolite rate of ALDH2*1/*1-encoded enzyme) completely inactive forms, respectively [37, 38]. The rs671 has been reported as an Asian-specific SNP, approximately 50% of Asians [39], including the Han Chinese in Taiwan [40, 41] carry ALDH2*1/*2 and ALDH2*2/*2 polymorphisms. This reduced ALDH2 enzymatic activity in East Asian populations has been reported to cause differences in susceptibility to many diseases, such as Asian flush [42] and a series of cancers [43].

Reviewing the crucial role of *ALDH2* in the dopamine metabolite pathway, and an association between *ALDH2* and dopamine receptor 2 (*DRD2*) has been reported in the BP-II without AD comorbidity [32]. In addition, the cognitive impairment caused by AD comorbidity in BP patients has been noted in the literature, the possible differences in metabolite accumulation caused by *ALDH2* gene variants. The effects on neuropsychological performance of the relationships between different *ALDH2* genotypes and comorbid ADs on neuropsychological performance in BP-II patients drawn from Han Chinese in Taiwan were proposed.

## Methods

This study has been approved by the Institutional Research Board (IRB) of National Cheng Kung University Hospital. Considering the ethnic effect, all the participants recruited in this study were unrelated and came from the Han Chinese population in Taiwan. All the participants were fully informed and made agreement to sign the informed consent forms.

### Participants

Patients were recruited from outpatient and inpatient settings, and were initially evaluated and diagnosed as BP-II by an attending psychiatrist. Afterwards each participant underwent a more detailed and structured interview with a clinical psychologist using the Chinese version of the Modified Schedule of Affective Disorder and Schizophrenia-Life Time (SADS-L) [44], with good inter-rater reliability [35], to verify that the diagnoses met the criteria of the DSM-IV-TR (Diagnostic and Statistical Manual of Mental Disorders, Fourth Edition, Text Revision) and to identify the healthy controls. Patients with DSM-IV Axis I diagnoses other than BP-II and AD, concomitant medical illnesses, borderline personality disorder, neurological disorders or brain injury, and lifetime illegal substance or alcohol use disorders (abuse or dependence) were excluded. The severity of mood symptoms was assessed using the Young Mania Rating Scale (YMRS) [45] and the Hamilton Depression Rating Scale (HDRS) [46, 47]. The criteria we used for BP-II diagnosis was 2 days for hypomania instead of 4 days, since an increasing validated support in other studies [48]. For the healthy controls (HCs) recruited from community via advertisement, all participants were reviewed by a well-trained research assistant to screen out if they have any psychiatric disorders. There were 323 participants: 193 with BP-II [86 males, 107 females], 86 of whom had comorbid AD (BP-II^+AD^), 107 of whom did not (BP-II^−AD^), and 130 healthy controls (BP^−^ and AD^−^) [77 males, 53 females]).

### Genotype data: Blood samples and genotyping

Each participant was drawn for 20 milliliters of venous blood, and their DNA was extracted from the lymphocytes. PCR amplification for SNPs (rs671) was performed in 10μl reactions with 5 ng of template DNA, 1×TaqMan SNP Genotyping Master Mix (Applied Biosystems, Foster City, CA), 20×probe (TaqMan SNP Genotyping Assays ID: C__11703892_10), and H_2_O. Thermal cycling initiated with a first denaturation step of 10 min at 95°C, followed by 40 cycles of denaturation at 95°C for 15 seconds and annealing at 60°C for 1 min. The allele-detection process was performed for 1 min at 60°C on a StepOnePlus Real-Time System (Applied Biosystems, Foster City, CA) to determine the allelic discrimination. A polymerase chain reaction and restriction fragment length polymorphism (PCRRFLP) analysis was used to determine the genotypes of *ALDH2*. The single nucleotide functional polymorphism sites in exon 12 of the ALDH2 gene were genotyped using protocols described elsewhere [49]. The laboratory technician who did the genotyping and read out the genotype data was blinded to the patients’ clinical data.

### Neuropsychological tasks

#### Continuous Performance Test

Conner’s Continuous Performance Test (CPT) [50] has been demonstrated as a measurement in which the participants need to maintain their focus attention with executive control to inhibit their response to distractors. The following indices have been reported as major measurements by CPT: [1] Inattentive indices: Errors of omission index measuring the errors of participants fail to respond to the target (OmeT); Commission errors (ComeT) are made when responses are given to non-targets; Hit reaction time (HRT) represents mean response time (milliseconds) for all target responses; Variability of Standard Error is a measure of response speed consistency; Detectability (*d*′) represents the ability of discriminating between targets and non-targets, and HRT Inter-Stimulus Interval (HRT ISI Change) examining changes in average reaction times across the different inter-stimulus intervals; Standard Error by Inter-Stimulus Interval (Hit SE ISI Change) examines change in the standard error of reaction times at the different Inter-Stimulus Intervals. [2] Vigilance indices: HRT by Block (HRT Block Change) measures changes in reaction time across the duration of the test. High scores indicate a substantial slowing in reaction times; Hit RT Block Change (HRT Block Change); [3] Impulsive indices include HRT standard error (HRT SE), representing the consistency of response times and expresses the SE response to targets, and Errors of commission index measuring the rate that participants respond to a non-target (X).

#### Wisconsin Card Sorting Test

The Wisconsin Card Sorting Test (WCST) has been reported as a measurement in certain types of executive functions: categorization, abstract reasoning, maintaining sets, set switching, strategic planning, and modulation of impulsive responding [51]. Several cognitive functions, including attention, working memory and visual processing are involved in performing WCST. The inter-rater reliability is 0.88–0.93, within-rater reliability is 0.91–0.96, and test-retest reliability is 0.57. The scored indices of WCST based on the total number of errors (TNE), perseverative errors (PE), conceptual level responses (CLRs), number of categories completed (NCC), and number of trials needed to complete the first category (TCC).

#### Wechsler Memory Scale-Third Edition

The Wechsler Memory Scale-Third Edition (WMS-III) [52], the most commonly used memory function test, consists of 18 subtests and produces eight composite index scores covering immediate and delayed recognition and recall of both auditory and visual stimuli. Composite scores were calculated for the eight standardized primary indices: Auditory Immediate (AIM), Visual Immediate (VIM), Immediate Memory (IM), Auditory Delayed (ADM), Visual Delayed (VDM), Auditory Recognition Delayed (ARDM), General Memory (GM), and Working Memory (WM).

### Statistical analyses

Pearson χ^2^ analysis was used to examine the gender differences and other categorical variables. Normality tests were performed to explore whether the variables conformed to parametric assumptions; however, not all variables conformed to parametric assumptions, non-parametric tests were conducted for the following comparisons. Significance was set at *p* < 0.05, and False Discovery Rate (Benjamini & Hochberg, 1995) was applied for the multiple corrections. SPSS 20.0 was used for statistical analysis.

## Results

### Group characteristics

The test of normality showed that age and educational level did not follow the normal distribution, non-parametric analyses were conducted to compare the demographic data among groups, the results showed no significant differences of age and educational level (*ps>*.05). But for the gender distribution, the results showed significant distribution in gender among three groups. Post hoc analysis showed significantly fewer males in the BP-II^+AD^ group than in the HC group (χ^2^ = 3.94, *p* = 0.047). Significantly fewer females were recruited in the HC groups, but gender distribution was not significantly different between the BP-II^+AD^ and BP-II^**−**AD^ groups (Table 1).

**Table 1:** Demographic data comparisons between patient groups and healthy controls.

Groups	BP-II^+AD^ (n = 86)	BP-II^–AD^ (n = 107)	HC (n = 130)	χ^2^ (*p*)
Gender (Male/Female)	36/50	49/58	77/53	7.08 (*0*.*03*)
Age (years)	34.84 ± 13.04 (33.00)	34.98 ± 13.55 (33.00)	31.80 ± 8.01 (30)	0.02 (0.88)^a^
Education level (years)	13.39 ± 3.32 (13.50)	12.99 ± 3.32 (12.00)	15.33 ± 1.65 (16)	1.29 (0.26)^a^
Age at BP onset (years)	14.28 ± 3.72 (14.00)	15.13 ± 5.12 (14.00)	--	1.16 (0.25)^a^
Duration of BP illness (years)	19.81 ± 11.96 (17.00)	19.02 ± 13.03 (15.00)	--	-0.90 (0.37)^a^
Age at AD onset (years)	19.49 ± 12.84 (17.00)	--	--	--
Duration of AD (years)	14.29 ± 12.96 (11.00)	--	--	--
HDRS score	16.91 ± 3.34 (18.00)	15.22 ± 4.61 (16.00)	--	-2.13(0.03)^a^
YMRS score	12.34 ± 3.46 (13.00)	13.38 ± 3.04 (14.00)	--	2.12(0.03)^a^
AD subtype (n)				
* GAD*	56	--	--	--
* Panic disorder*	19	--	--	--
* OCD*	7	--	--	--
* Social phobia*	15	--	--	--
* Specific phobia*	20	--	--	--
* PTSD*	2	--	--	--
Number of comorbid Ads				
* 1*	61	--	--	--
* ≥ 2*	25	--	--	--

Data represented as Mean±SD (median).

^a^Non-parametric comparisons

### Clinical characteristics: Severity and duration of BP

HDRS scores were significantly higher in the BP-II^**+**AD^ group than in the BP-II^**−**AD^ group, but YMRS scores were significantly lower in the BP-II^**+**AD^ group than in the BP-II^-AD^ group. The duration of illness; however, was not significantly different between the patient groups (Table 1).

### Neuropsychological performance

Because not all variables conformed to parametric assumptions, non-parametric analyses were conducted, Dunn post hoc comparisons were conducted for Kruskal-Wallis test and False Discovery Rate (Benjamini & Hochberg, 1995) was applied for the multiple corrections. Overall, the patient groups performed significantly less well on neuropsychological tasks than did the HC group in most domains (Tables 2 and 3).

**Table 2:** Neuropsychological performance in attention and executive function between patient groups and healthy controls.

Groups	BP-II^+AD^ (n = 86)	BP-II^–AD^ (n = 107)	HC (n = 130)	χ^2^ (*p*)	Post-hoc^a^
Continuous Performance Test (CPT)					
* Omission T-score*	110.83 ± 107.64 (62.00)	103.12 ± 106.49 (59.00)	46.77 ± 10.81 (44.87)	100.76 (< .0005)	HC> **BP-II^+AD^; **BP-II^-AD^
* Commission T-score*	56.81 ± 12.93 (55.00)	58.23 ± 11.40 (57.00)	40.08 ± 10.84 (46.17)	48.05 (< .0005)	HC> **BP-II^+AD^; **BP-II^-AD^
* HRT T-score*	58.23 ± 17.26 (56.00)	53.16 ± 15.42 (52.47)	45.85 ± 10.24 (43.96)	42.26 (< .0005)	HC> **BP-II^+AD^; **BP-II^-AD^
* HRT SE T-score*	68.49 ± 25.06 (62.50)	61.78 ± 22.76 (56.00)	42.82 ± 10.71 (40.93)	93.13 (< .0005)	HC> **BP-II^+AD^; **BP-II^-AD^
* Variability T-score*	65.06 ± 18.17 (61.82)	61.96 ± 19.30 (58.00)	44.76 ± 10.03 (42.71)	93.76 (< .0005)	HC> **BP-II^+AD^; **BP-II^-AD^
* Detectability (d′) T-score*	54.09 ± 10.87 (54.00)	55.09 ± 9.52 (56.08)	48.23 ± 10.10 (48.95)	32.23 (< .0005)	HC> **BP-II^+AD^; **BP-II^-AD^
* Perseverations % T-score*	86.28 ± 71.34 (60.14)	80.19 ± 63.80 (54.98)	47.96 ± 9.82 (45.77)	82.25 (< .0005)	HC> **BP-II^+AD^; **BP-II^-AD^
* HRT Block Change T-score*	55.93 ± 15.88 (53.00)	57.66 ± 15.63 (54.84)	50.98 ± 8.87 (49.79)	14.61 (.001)	HC> *BP-II^+AD^; **BP-II^-AD^
* HRT SE Block Change T-score*	60.94 ± 15.79 (57.92)	64.37 ± 18.20 (62.00)	55.36 ± 10.63 (54.65)	19.05 (< .0005)	HC> *BP-II^+AD^; **BP-II^-AD^
* HRT ISI Change T-score*	53.95 ± 17.03 (49.16)	57.63 ± 71.25 (49.00)	49.42 ± 9.53 (49.31)	2.23 (0.33)	--
* Hit SE ISI Change T-score*	53.48 ± 19.24 (52.74)	52.67 ± 16.85 (50.00)	48.51 ± 10.70 (46.51)	6.41 (0.04)	HC> *BP-II^+AD^
Wisconsin Card Sorting Test (WCST)					
* Total number correct (TNC)*	87.09 ± 21.24 (91.00)	88.31 ± 20.20 (93.00)	97.59 ± 16.48 (103.00)	27.64 (< .0005)	HC> **BP-II^+AD^; **BP-II^-AD^
* Total number of errors (TNE)*	39.67 ± 19.71 (37.00)	38.52 ± 19.03 (33.00)	30.41 ± 16.48 (25.00)	25.12 (< .0005)	HC> **BP-II^+AD^; **BP-II^-AD^
* Perseveration error (PE)*	22.91 ± 16.18 (18.00)	22.92 ± 17.31 (18.00)	15.85 ± 10.65 (12.50)	29.34 (< .0005)	HC> **BP-II^+AD^; **BP-II^-AD^
* Number of completed categories (NCC)*	5.56 ± 3.01 (6.00)	5.61 ± 2.66 (6.00)	7.41 ± 2.46 (8.00)	36.03 (< .0005)	HC> **BP-II^+AD^; **BP-II^-AD^
* Trials to complete the first category (TCC)*	19.23 ± 18.03 (12.00)	17.19 ± 10.58 (12.00)	17.56 ± 13.05 (12.00)	0.21 (0.9)	--
* Conceptual level (CL)*	0.68 ± 0.41 (1.00)	0.73 ± 0.37 (1.00)	0.71 ± 0.18 (0.02)	17.37 (< .0005)	HC> *BP-II^+AD^; **BP-II^-AD^

**Table 3:** Neuropsychological performance in memory between patient groups and healthy controls.

Groups	BP-II^+AD^ (n = 86)	BP-II^–AD^ (n = 107)	HC (n = 130)	χ^2^ (p)	Post-hoc^a^
Wechsler Memory Scale (WMS-III)					
* Auditory Immediate Index*	104.59 ± 67.35 (100.00)	99.22 ± 16.52 (100.00)	106.81 ± 13.57 (105.00)	21.32 (< .0005)	HC > **BP-II^+AD^; **BP-II^-AD^
* Visual Immediate Index*	95.84 ± 18.27 (97.00)	96.20 ± 16.01 (97.00)	100.21 ± 14.59 (103.00)	5.14 (0.08)	--
* Immediate Memory Index*	96.05 ± 16.91 (94.00)	97.79 ± 16.44 (100.00)	104.33 ± 12.32 (104.00)	17.95 (< .0005)	HC > **BP-II^+AD^; *BP-II^-AD^
* Auditory Delay Index*	96.46 ± 17.61 (100.00)	99.01 ± 17.73 (102.00)	106.98 ± 13.11 (108.00)	25.31 (< .0005)	HC > **BP-II^+AD^; **BP-II^-AD^
* Visual Delay Index*	94.16 ± 18.01 (94.00)	96.88 ± 18.69 (97.00)	98.93 ± 13.96 (100.00)	2.69 (0.26)	--
* Auditory Delayed Recognition Index*	96.82 ± 16.72 (95.00)	97.29 ± 17.70 (100.00)	106.98 ± 12.88 (110.00)	26.17 (< .0005)	HC > **BP-II^+AD^; **BP-II^-AD^
* General Memory Index*	95.01 ± 17.47 (97.00)	97.76 ± 17.33 (100.00)	104.40 ± 13.16 (104.00)	19.50 (< .0005)	HC > **BP-II^+AD^; *BP-II^-AD^
* Working Memory Index*	97.14 ± 15.71 (97.00)	93.78 ± 13.16 (94.00)	104.67 ± 15.17 (106.00)	37.04 (< .0005)	HC > **BP-II^+AD^; **BP-II^-AD^

Data represented as Mean±SD (Median); χ^2^ represents Kruskal-Wallis test result;

^a^Dunn post hoc comparisons

* *p <* .*05*; ** p-value < 0.002 (corrected significance level, controlled False Discovery Rate, FDR)

HC: healthy controls; BP-II: bipolar II disorder; AD: comorbid anxiety disorder; GAD: generalized anxiety disorder; OCD: obsessive-compulsive disorder; PTSD: post-traumatic stress disorder.

To reduce type I error, factor analysis with principal component analysis (PCA) was used to condense neuropsychological measurements to several composite scores, six factors with eigenvalues > 1.00 from the varimax rotation were retained: Factor 1 was a composite score for WCST, factor 2 was inattentiveness for CPT, factor 3 was distractibility for CPT, factor 4 was vigilance for CPT, factor 5 was composite memory for WMS and factor 6, auditory immediate memory for WMS. Kruskal-Wallis tests showed significant differences on all composite scores (*ps* < .0005), except the factor 4 for CPT, vigilance (*p* = .04) after multiple corrections (*p* = .008, Bonferroni correction). Dunn post hoc showed that HCs significantly performed better on all cognitive composited domains than did both patient groups (*ps* < .0005), but for the CPT factor, vigilance, post hoc showed significance only between the BP-II^+AD^ group and the HC (*p* = .03). However, no significant difference was found between two patient groups, except the factor 3 distractibility, that the BP-II^+AD^ showed poor distractibility compared to the BP-II^-AD^. Moreover, the BP-II^+AD^ group was found to have significantly poor attentiveness than the BP-II^-AD^ (*p* = .03).

To study the effect of ALDH2 polymorphisms on neuropsychological performance, the ALDH2*1/*2 + *2/*2 genotypes were combined into a single group for data analysis due to small numbers of patients 10 (5.2%) had the ALDH2*2/*2 genotype. The numbers of ALDH2*1/*1 and ALDH2*1/*2+ALDH*2/*2 for the BP-II with AD group were 40 and 46 respectively, and for the BP-II without AD group were 59 and 48. We found that there was no significant difference of *ALDH2* polymorphism frequency between groups of BP-II^**+**AD^ and BP-II^**–**AD^ (χ^2^ = 1.42, *p* = .23).

Mann-Whitney analyses were conducted in BP-II (^+AD^ and −^AD^) groups to compare the effect of *ALDH2* polymorphisms on composite scores of neuropsychological battery. The results showed no significant differences on composite cognitive scores between different *ALDH2* polymorphisms in the HCs. (*ps*>.05). In addition, to study the correlation between AD comorbidity and *ALDH2* polymorphisms in the BP-II, we found that in the BP-II^**+**AD^, participants carried ALDH2*1/*1 significantly had greater score on the factor 2, for the CPT inattentiveness than did those carried the ALDH2*1/*2 + *2/*2 genotypes. Moreover, in the BP-II^**−**AD^ group, patients carried ALDH2*1/*1 genotype significantly performed worse on the factor 5 for WMS memory system than did those carried the ALDH2*1/*2+ALDH2*2/*2 genotypes (Table 4). Moreover, Spearman ρ correlation showed significant relationship between *ALDH2* polymorphisms and factor 2 for CPT inattentiveness in BP-II^+AD^ (ρ = −.22, *p* = .04) and factor 5 for WMS composite memory score (ρ = .20, *p* = .039). Further, stepwise linear regression with *ALDH2* gene variants and AD comorbidity as independent factors showed that *ALDH2* gene variants significantly were correlated to the factor 5 for WMS memory system in the BP-II^-AD^ group (Adjusted R^2^ = .04; F = 5.01, p = .027).

**Table 4:** Comparisons of neuropsychological performance between BP-II groups stratified by the *ALDH2* polymorphism.

Groups	BP-II^+AD^ (n = 40/46)		χ^2^ *(p)*	BP-II^–AD^ (n = 59/48)		χ^2^ *(p)*
	ALDH2/*1/*1	ALDH2/*1/*2+ ALDH2/*2/*2		ALDH2/*1/*1	ALDH2/*1/*2+ ALDH2/*2/*2	
Age at BP onset (years)	14.93 ± 4.02 (14.00)	13.67±3.34 (14.00)	-1.51 (0.13)	15.36± 5.00 (15.00)	14.87± 5.30 (14.00)	-1.69 (0.09)
Duration of BP illness (years)	20.90 ± 12.54 (17.50)	18.79±11.45 (15.00)	-0.58 (0.56)	19.85±13.37 (17.00)	18.07±12.71 (11.50)	-1.55 (0.12)
Age at AD onset (years)	21.33 ± 13.21 (18.00)	17.88±12.55 (15.00)	-1.30 (0.19)	--	--	--
Duration of AD (years)	13.36 ± 12.63 (10.50)	15.07±13.32 (11.00)	0.64 (0.53)	--	--	--
HDRS scores	16.43 ± 3.40 (18.00)	17.33±3.26 (18.00)	0.95 (0.34)	15.88±4.39 (16.00)	14.42±4.79 (15.50)	-0.94 (0.35)
YMRS scores	11.48 ± 3.61 (12.50)	13.09±3.17 (14.00)	2.00 (0.045)	13.69±3.17 (14.00)	13.00±2.87 (14.00)	-0.53 (0.59)
Factors for neuropsychological tests						
* Factor 1 for WCST*	-0.34±1.23 (-0.17)	-0.21±1.08 (0.12)	0.46 (0.64)	-0.21±1.08 (0.11)	-0.12±1.04 (0.13)	0.34 (0.74)
* Factor 2 for CPT*, inattentivenes	0.69±1.20 (0.44)	0.24±1.31 (0.23)	**-2.04 (0.04)**	0.24±1.31 (-0.25)	0.06±0.79 (-0.04)	0.08 (0.94)
* Factor 3 for CPT*, *distractibility*	0.26±1.12 (0.04)	0.43±0.98 (-0.07)	-0.84 (0.40)	0.43±0.98 (0.60)	0.13±0.83 (0.18)	-1.86 (0.06)
* Factor 4 for CPT*, *vigilance*	-0.13±1.29 (-0.34)	0.25±1.03 (-0.10)	0.95 (0.34)	0.25±1.03 (-0.05)	0.19±1.32 (0.06)	-0.24 (0.81)
* Factor 5 for WMS*, *memory*	-0.22±1.46 (-0.06)	-0.27±1.00 (-0.16)	-0.86 (0.39)	-0.27±1.00 (-0.25)	0.16±0.96 (0.01)	**2.05 (0.04)**
* Factor 6 for WMS*, *auditory immediate memory*	0.12±1.91 (-0.14)	-0.34±0.77 (-0.21)	-1.05 (0.30)	-0.34±1.01 (-0.23)	-0.20±0.82 (-0.15)	0.84 (0.40)

Data represented as Mean±SD (Median); BP-II: bipolar II disorder; AD: anxiety disorder

Subtypes of AD might have different effects on neuropsychological performance. Most of AD^**+**^ patients in the BP-II^**+**AD^ group had GAD; thus, we then divided BP-II^**+**AD^ into GAD^**+**^ and GAD^**−**^ subgroups. However, no significant differences of AD subtypes on neuropsychological performance were found (*ps* > 0.05).

## Discussion

### Group characteristics

Both the BP-II groups reported fewer years of education compared to the HC group, but, this difference did not reach statistical significance due to skewed distribution. This tendency may reflect the onset of BP during the patient’s teenage years and interrupt their receiving education [53]. Patients with BP-II are frequently dominated by depressive symptoms, which often lead to a misdiagnosis [54]. The BP-II^**+**AD^ group had significantly higher HDRS scores than did the BP-II^**−**AD^ group, which may reflect one effect of AD on BP-II, especially when the patient is in a depressive state [55–57]. Moreover, we found a higher proportion of BP-II^**+**AD^ with GAD than any other form of AD, which agrees with previous report by Chang et al. (2012). It seems to support the notion that BP-II is derived from major depressive disorder (MDD), which is commonly comorbid with AD [58, 59].

### Neuropsychological performance

Both the BP-II^**+**AD^ group and the BP-II^**−**AD^ group performed significantly less well than did the HC group on attention, memory, and executive function tests, but there were no significant differences between the BP-II groups. The effect of comorbidity of AD was not found in the current study; this was inconsistent with previous report by Wu et al. (2011). One explanation might be a small sample compared in Wu et al.’s study, and that there were not enough AD subtypes for a broader generalization. Moreover, no significant correlation between AD and WCST indices in the BP-II groups which was consistent with Airaksinen et al. (2009) [60] but not with Wu et al. (2011) and Boldrini et al. (2005) [61]. It might be that most comorbid AD subtypes in our study were GAD and specific phobia showing no effect on executive dysfunctions [60]. Subtypes of ADs might affect cognitive dysfunction profiles [62] and need further investigation. Another possibility is that all of our enrolled patients had taken medication that concealed their cognitive dysfunction, and that the medication had a greater effect than did the AD [63]. Additional research on the different effects of BP-II and AD is needed to clarify this.

Although we found some marginal interaction between AD and *ALDH2* genotypes in sustained attention system and memory system, which indicated a possible protective factor of ALDH2*1/*2 and ALDH2*2/*2 on sustained attention system in BP-II patients from decreasing sustained attention while having AD comorbidity. In addition, a possible protective factor of ALDH2*1/*2 and ALDH2*2/*2 is for some of memory functions in the BP-II^**−**AD^ from the effect of bipolar disorder on decreasing memory functions.

The interaction between *ALDH2* gene variants and AD comorbidity could be that ALDH2*1/*1 promote the effect of AD on attentional impairment in the BP-II. Dopamine has been linked with attention and impulsivity [64], and it interacts with other powerful brain chemicals and neurotransmitters (e.g., serotonin and the opioids), which are associated with mood control [65]. The relationship between the dopamine metabolized enzyme and sustained attention system was noticed. In BP-II^**+**AD^, patients who carried the ALDH2*1/*1 genotype had significantly higher levels of inattention and impulsivity, which is associated with the dopamine system. Patients with BP-II^**−**AD^ who carried the ALDH2*1/*1 genotype performed less well on the memory system than did those who carried the ALDH2*1/*2 and ALDH2*2/*2. The dopamine circuit is also linked with frontal-subcortical or mesolimbic circuitry [23], which is related to mood dysregulation and memory functions in some subcortical regions, such as the hippocampus and amygdala [24–26]. The interaction between dopamine metabolize pathway and major mental disorder, such as bipolar disorder needs further investigation.

To the best of our knowledge, so far no studies focusing on the association between *ALDH2*-encoded dopamine metabolic enzymes and cognitive performance in studying the effect of comorbidity in BP-II patients. Previously, the *ALDH2* polymorphisms have been implied to be related to Alzheimer’s disease [66] which implied an association between *ALDH2* polymorphisms and memory system; however, in an older Korean community, Kim et al. (2004) [67] found no significant association between *ALDH2* polymorphisms and cognitive outcome, using Mini Mental State Examination (MMSE). The MMSE although has been reported as a measurement tool to screen for dementia, it might not contain variety of cognitive functions and limit the generalization. More recently, an association between *ALDH2* gene variants between language and attention dysfunction was noticed in patients with Parkinson’s disease (PD) [68]. Yu et al. (2016) reported that PD patients with inactive *ALDH2*-encoded enzyme had worse attention and language functions compared to those carrying ALDH2*1/*1. Lee et al. (2017) studied in the effect of *ALDH2* polymorphisms on cytokines and cognitive correlation in treating patients with bipolar disorder. They found that a significant associations between ALDH2*1/*1 genotype and the improvement of cognitive function in patients with bipolar disorder in a one-year follow up.

The cognitive impairment profiles in mental disorders, such as bipolar disorder are heterogeneous, and the effect of comorbidity varied in previous findings. The possible association between *ALDH2* polymorphisms and comorbidity in bipolar disorders on cognitive profiles needs further investigation. The findings in the current study may be not significant enough for the generalization due to multiple cognitive tests were compared; our finding could provide preliminary information in such aspect for further investigations.

This study has some limitations. First, because our sample was small, no specific ADs were separated into subgroups for comparison. Patients with BP-II^**+**AD^ were divided into GAD^**+**^ and GAD^**−**^ subgroups to compare their cognitive performance, but the differences were not significant. Although, Kim et al. (2014) [69] hypothesized that obsessive-compulsive disorder (OCD) had a consistently negative effect on the outcome of BP-I, regardless of clinical situation; not many BP-II patients with comorbid OCD in this study, we could not draw any conclusions about OCD’s effect on BP-II. Secondly, educational level was not matched between HCs and patient groups which may limit our findings generalization. This could be that BP-II patients had onset during their teenagers and limited their receiving education. Third, our HC participants were recruited as a reference group, but we had no control group of pure AD participants. Thus, our findings should be interpreted with caution. Finally, it is also possible that whether the patients were having a mood episode or in remission or in an inter-episodic state affected their cognitive performance.

## Supporting information

S1 File ALDH2 on cognition between BPII with and without AD.sav. Genotype and cognitions for comorbidity for bipolar disorders.
(SAV)
